# Gleanings in Science and Medicine

**Published:** 1893-07-15

**Authors:** 


### Gleanings in Science and Medicine.

Recent experiments on the effect of low temperatures on chemical action are of considerable interest, and will doubtless be still further developed. Pictet claims to have established the fact that below a certain temperature chemical affinity ceases to act. Diluted sulphuric acid, solidifying at 56 deg., combined at 125 deg. with powdered caustic soda, strongly compressed, produced no chemical action. The temperature was raised to 80 deg., when chemical action not only began, but was so powerful as to burst the vessel in which the substances were contained. Again, common salt and sulphuric acid do not re-act at a lower temperature than 25 deg. The general result of Pictet's investigations, which are well worth studying in detail, appears to be that he has come to the conclusion that chemical action is impossible at a temperature of between 125 deg. and 150 deg.

The effect on health of going barefooted has lately been discussed in some of the newspapers. It is pointed out that in no other part of the body are there so many nerve endings as in the sole of the foot, and that the sciatic nerve, passing from the spinal cord through the leg, branches out in the foot in close contact with the earth; and it has been argued that this is meant, in the economy of nature, to provide some electrical connection with "mother earth," which may have some important physiological results. Therefore, it is said, we should not interfere with this natural contact with the earth. After all, however, whether or not we ought to go barefooted, we shall never do it, so long as we can find the means of protecting our feet from the mud and cold of winter, and the dust and thorns of summer. But as to the healthiness of going without shoes or stockings there can be no question. Some of the healthiest children in the world are to be found in the Scottish Highlands, where shoes are seldom worn at an earlier age than twelve or thirteen. The negro and coolie labourers, who work barefooted, are usually in robust health. Brown, in the "History of Man," tells of an African monarch who suffered from what appears to have been a chronic cold in his head, besides other ailments, while his people were always as well as possible. Can it be that the reason was that, by the laws of his kingdom, he alone was permitted to clothe his feet, and that he gratified his vanity by always wearing gorgeous sandals? It is probably generalising too much to state, as a medical fact, that the barefooted races are the healthiest. But it is certain that bare feet are healthier than badly shod feet. In our English villages children are constantly sent to school in wet weather with holes in their shoes. They sit for hours with damp feet, and illnesses are the result. If their parents would send them off barefooted, as is done in Scotland and Ireland, their feet would dry by evaporation in a short time, and it would be found that no harm ever came from walking through pools of rain water. But how long would it take to persuade the English villager of this?

The Japs for the most part are a non-meat eating race; indeed, in Central Japan and in out-of-the-way parts the inhabitants have never tasted such food, a fact partly due, no doubt, to economic reasons, a meat diet being an expensive luxury in the far East. Statistics of a recent year go to prove that out of the 1,021,503 head of cattle existing in the country, only the comparatively small number of 84,711 were slaughtered for purposes of food. The tendencies of Japan being thus inclined towards vegetarianism, a case of a positively inhuman exception to this state of things is all the more astonishing. It appears that quite recently in the Prefecture of Meji a native was discovered digging up the body of a newly-buried child, with the intent to eat its flesh. Upon arrest, the man pleaded in self-defence that he had been led to believe that human flesh would cure him of a disease from which he was suffering. A cure which all unforeseen cost the credulous believer a three months' residence in prison.

In describing the process of disinfection carried out by the Municipal Service in Paris, M. Rochard defends the use of the disinfectant medium, bichlorure of mercury, commonly employed, and claims that no accident has ever been observed among persons subsequently occupying places disinfected in this matter. Dr. de Fournes calls attention to the grave dangers which nevertheless may arise from the indiscriminate use of corrosive sublimate in the disinfection of household furniture, &c., since though undoubtedly every species of microbe is inevitably destroyed by it, it remains uncertain whether traces of mercury sufficient to produce symptoms of mercurial poisoning may not remain behind even months after. It is very doubtful whether all traces of mercury can be removed even by copious washing, and among certain persons imponderable atoms suffice to produce disagreeable symptoms. It is urged that a serious inquiry should be undertaken with a view to ascertaining whether accidents may not occur after the use of corrosive sublimate during a period extending over several months.

